# Timescales of Evidence Evaluation for Decision Making and Associated Confidence Judgments Are Adapted to Task Demands

**DOI:** 10.3389/fnins.2020.00826

**Published:** 2020-08-13

**Authors:** Rashed Harun, Elizabeth Jun, Heui Hye Park, Preetham Ganupuru, Adam B. Goldring, Timothy D. Hanks

**Affiliations:** Department of Neurology and Center for Neuroscience, University of California, Davis, Davis, CA, United States

**Keywords:** perceptual decision, change detection, behavioral model, confidence, audition, evidence evaluation

## Abstract

Decision making often involves choosing actions based on relevant evidence. This can benefit from focussing evidence evaluation on the timescale of greatest relevance based on the situation. Here, we use an auditory change detection task to determine how people adjust their timescale of evidence evaluation depending on task demands for detecting changes in their environment and assessing their internal confidence in those decisions. We confirm previous results that people adopt shorter timescales of evidence evaluation for detecting changes in contexts with shorter signal durations, while bolstering those results with model-free analyses not previously used and extending the results to the auditory domain. We also extend these results to show that in contexts with shorter signal durations, people also adopt correspondingly shorter timescales of evidence evaluation for assessing confidence in their decision about detecting a change. These results provide important insights into adaptability and flexible control of evidence evaluation for decision making.

## Introduction

In statistical analysis, change point detection refers to the identification of the times when the underlying probability distribution of a time series changes. This has important applications in manufacturing, medical diagnosis, economics, and many other domains. It is also an important component of everyday human decision making. Many decisions require or benefit from explicit detection of changes in the environment. These range from situations as mundane as determining when to go at a traffic light to situations as critical as determining when to flee from potential danger.

The ideal observer decision process for change point detection differs from many other types of decisions that benefit from perfect integration of evidence. Perfect integration combines separate samples of evidence by giving them equal weight. For a stable environment, equal weighting of independent samples of evidence will average out variability to obtain the best estimate of the environmental state to inform one’s decision. This is the basis of accumulation to bound decision models, including the sequential probability ratio test (SPRT) and drift-diffusion model (Wald and Wolfowitz, 1948; Stone, 1960; Laming, 1968; Link, 1992; Ratcliff and Rouder, 1998; Ratcliff and Smith, 2004; Palmer et al., 2005).

In contrast, change point detection inherently involves environmental instability and comparison of the environmental state at different times. In this situation, combining all samples of evidence together with equal weight can reduce sensitivity. In the extreme, imagine a scenario where failure to detect a change in a timely manner results in a highly salient secondary cue that the change has been missed. For example, if you are stopped at a busy traffic intersection waiting for the light to turn green, failure to move your car within a few seconds of that change will likely be cued by honking horns from fellow drivers. Thus, more weight should be given to recent samples of evidence about the light’s color because it is unlikely the light turned green in the more distant past if you have not heard honking horns. In general, because perfect integration of evidence gives equal weight to all time points, it can result in a loss of sensitivity for change point detection where the most relevant part of the signal occurs in a limited window of time (Lasley and Cohn, 1981).

Solutions to this problem universally involve differential weighting of evidence over time for optimal change point detection. For example, adopting “leaky” rather than perfect integration of evidence introduces a time constant of evidence decay, and therefore gives more weight to recent evidence compared to that gathered earlier in time (Ossmy et al., 2013; Glaze et al., 2015). This and related solutions effectively reduce the timescale of evidence evaluation compared to perfect integration.

The optimal timescale of evidence evaluation for change point detection depends on the statistics of the environmental changes (Ossmy et al., 2013; Glaze et al., 2015; Radillo et al., 2017; Piet et al., 2018). Shorter timescales are required for detecting changes in more volatile environments and when signal durations are briefer. Humans have been shown to adapt their timescale of evidence evaluation accordingly as a function of the distribution of signal durations and timing for visual stimuli (Tsetsos et al., 2012; Ossmy et al., 2013; Bronfman et al., 2016; Levi et al., 2018). Humans have also been shown to adapt their timescales of evidence evaluation in the optimal direction as a function of volatility when classifying source distributions for the location of visual stimuli (Glaze et al., 2015). Additionally, both humans and rats have been shown to adapt their timescales of evidence evaluation in the optimal direction as a function of volatility when discriminating the current state of a sensory stimulus in a changing environment (Glaze et al., 2015; Piet et al., 2018). Furthermore, for this work in rats, the timescale of evidence evaluation has been shown to be optimal when taking into account noise in sensory processing (Piet et al., 2018).

Here, we extend this line of work using an auditory change detection task that we have recently developed (Johnson et al., 2017). We corroborate previous findings that the timescales of evidence evaluation can be adapted to expected signal durations (Ossmy et al., 2013), and extend those results beyond the visual domain to the auditory domain. In addition to using model-based analyses similar to the previous work, we demonstrate this result using model-free psychophysical reverse correlation (RC) methods. Our task also involves a confidence judgment component (Ganupuru et al., 2019), allowing us to show that the timescale of evidence evaluation for confidence in the detection responses also adapts to expected signal durations. Our results suggest that adaptive timescales of evidence evaluation are a general feature of decision processes for change point detection.

## Materials and Methods

### Subjects

A total of 12 subjects were recruited to perform the experiments. One subject discontinued experimentation at her will. We analyzed the data from 11 subjects (eight female and three male). All subjects were aged 18–32 and members of UC Davis. Three subjects had knowledge about the research motivations prior to data collection, and the remaining subjects were naive. Subjects were compensated with $10 Amazon gift cards for each session that lasted approximately 60 min irrespective of task performance. Study procedures were approved by the UC Davis Institutional Review Board, and all subjects provided informed consent.

### Change Detection Task

#### Apparatus

The experimental apparatus consisted of three conical ports (left, middle, and right) fitted with LEDs to indicate when the port could be utilized during a trial and IR beam break sensors that detected when a finger was inserted into a port. The task was programmed in MATLAB, which controlled a BPOD interface that measured the outputs of the behavioral task in real-time (Sanworks). The auditory stimulus was generated using Pulse Pal (Sanders and Kepecs, 2014), while the feedback sounds were generated through the PC and played through headphones worn by the subjects.

#### Task Structure

**FIGURE 1:**
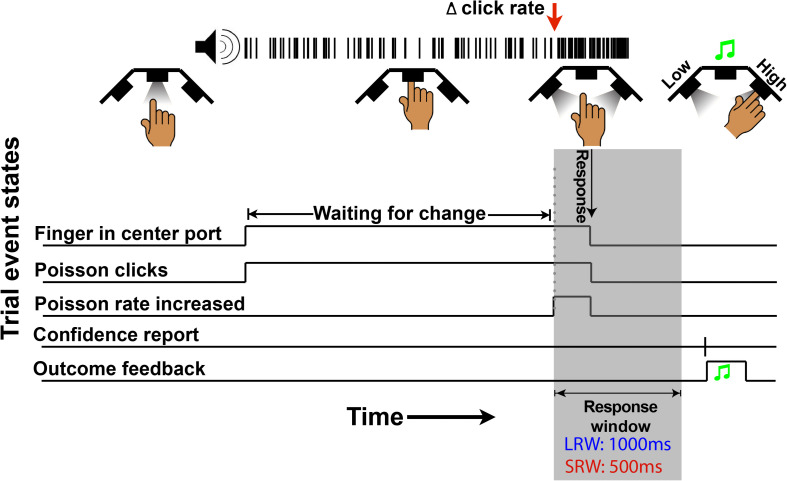
Schematic of a change trial in the auditory change detection task. Trials begin with the LED “on” in the center port, instructing subjects to insert their finger. Thereafter, a series of stochastic auditory clicks generated by a 50 Hz Poisson process play, followed by an increase in the generative click rate at a random time (red arrow in this example). For a correct response, subjects must withdraw their finger from the port within the allotted response window (gray region – 1,000 ms in the LRW condition and 500 ms in the SRW condition). Lastly, subjects have to report their confidence in successfully completing the trial by poking the right port or left port to indicate high or low confidence, respectively. Auditory feedback was then provided to indicate if subjects were correct.

The structure of the change detection task is a modified version of what was previously described (Ganupuru et al., 2019). Subjects began each trial by inserting their finger into the middle port of the behavioral apparatus (Figure 1), whereupon a train of auditory clicks played. The clicks were randomly generated through a Poisson process with a mean click rate of 50 Hz. Thereafter, in change trials, there was a randomly timed change in the generative click rate that subjects were tasked to respond to by removing their finger from the port within a response window. The change time was drawn from a truncated exponential distribution (mean 4 s, minimum 0.5 s, and maximum 10 s) to approximate a flat hazard function, meaning that the instantaneous probability of a change occurring does not increase or decrease throughout a trial. In a random 30% of trials, there was no change (catch trials), and the stimulus continued at a 50 Hz rate for a duration matching the response window if there had been a change. For catch trials, subjects were tasked to keep their finger inserted for the full duration of the stimulus. Thus, there were four possible trial outcomes. If the subject removed their finger when there was no change in the generative click rate, it was classified as a “false alarm” (FA). In catch trials, if the subject left their finger in the port for the duration of the stimulus, it was classified as a “correct rejection” (CR). In change trials, if the subject reported a change within the allotted response window, it was classified as a “hit,” while if it was not reported in time it was classified as a “miss.” Sometimes, subjects responded shortly after the response window elapsed, which could cause some ambiguity for the subject about whether they removed their finger within that response window or whether it was too late. For this reason, we incorporated a subtle 200 ms haptic feedback signal if the finger was removed during the stimulus presentation as in the case of a “hit” or FA but not a “miss” or CR.

In the last step of each trial, subjects had to indicate their confidence in successfully completing the trial by inserting their finger into the right port for high confidence or left port for low confidence. Subjects were instructed to report confidence in a hit if they had removed their finger in time (i.e., for a trial that could be either a hit or FA), and confidence in a CR if they had not removed their finger before the end of the stimulus (i.e., for a trial that could be either a CR or miss). Auditory feedback was delivered through the headphones to indicate whether the subject was successful in the trial regardless of indicated confidence, using a high frequency or low frequency fluttering tone for correct or incorrect, respectively. To encourage the reporting of confidence consistently within and across subjects, there was a point structure similar to previous work (van den Berg et al., 2016). If the subject indicated *high* confidence, they were awarded two points or lost three points if they were successful or unsuccessful, respectively. In contrast, if the subject indicated *low* confidence, they were awarded one point or lost one point if they were successful or unsuccessful, respectively. In order to maximize their score, subjects should indicate high confidence if they were >2/3 likely to be successful. After the confidence report, the inter-trial interval did not begin until a minimum time from trial start so that faster responses would not result in shorter overall trial times.

Subjects performed one session per day of this experiment, which consisted of three to four approximately 15-min experimental blocks separated by short breaks. For each experimental condition (see below) subjects performed one training session and additional training sessions as necessary to meet satisfactory performance criteria (<25% FA rate and accuracy >45% for the session). Thereafter, the subjects completed 3–5 sessions in each condition.

#### Experimental Conditions

This experiment was designed to examine how altering the response window affected the timescale of evidence evaluation. Thus, subjects were tested in two different explicitly instructed response window conditions – a long 1,000 ms response window condition (LRW) and a short 500 ms response window condition (SRW). Subjects were initially tested on the LRW and then the SRW version of the task. We used this design rather than counter-balancing because we wanted to first replicate our results from a previous manuscript that used a response window closer to the duration of the LRW condition (Ganupuru et al., 2019). This provides a foundation for observing changes in strategy relative to behavior that has already been characterized.

By virtue of having different response windows, changes of the same magnitude should be easier to detect in the LRW condition because subjects have more time to respond. To control for task difficulty, we manipulated the *proportions* of change magnitudes (Δs) in the two conditions. Subjects were exposed to the same set of Δs (10, 30, 50, and 70 Hz increases) in each condition; however, the low magnitude Δs (10 and 30 Hz) were more prevalent in the LRW, while the high magnitude Δs (50 and 70 Hz) were more prevalent in the SRW. Specifically, the Δs were randomly drawn from linear probability distributions. The probabilities for Δs 10, 30, 50, and 70 Hz in the LRW condition were *p* = 0.35, 0.28, 0.22, and 0.15 and in the SRW condition were *p* = 0.13, 0.21, 0.29, and 0.37. These distributions yield similar miss rates in the two conditions for an ideal observer after accounting for non-decision time (NDT) and while keeping FA rate the same.

We considered keeping proportions of Δs similar for the two conditions. That would have eliminated a confound in stimulus change magnitude between conditions but introduced a confound in task difficulty because it is more difficult to detect the same change in a shorter period of time. The latter could be particularly problematic for our experimental approach if it resulted in fewer FA trials that we use to estimate the psychophysical RC detection report kernel (see below). We also worried that this difference could lead to differences in motivation. To combat these concerns, we devised an alternative that would still use the same magnitudes of stimulus changes in the two conditions, but with proportions set so that the miss rate would be the same for an ideal observer who keeps FA rates matched between conditions. In this way, we can directly compare performance on matching stimulus change magnitudes while keeping overall difficulty the same.

### Data Analysis

#### Behavioral Performance

**FIGURE 2:**
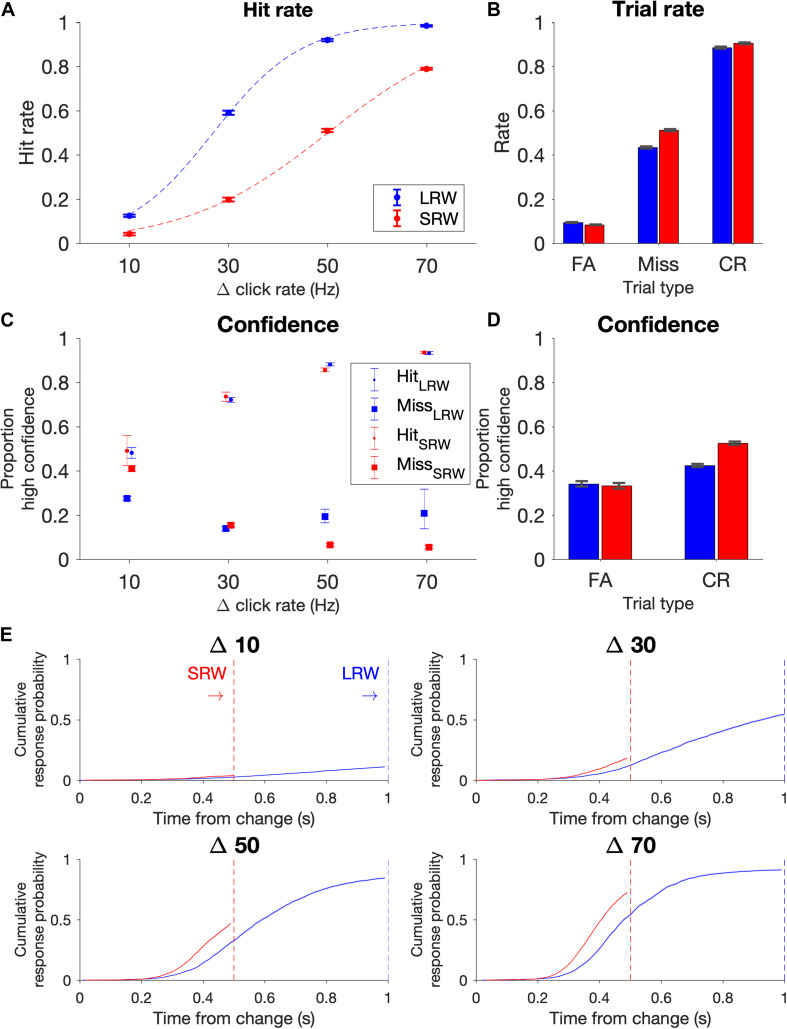
Task performance (combined *N* = 11 subjects). **(A)** Psychometric functions on the task conditions as delineated by hit rate vs change magnitude. Data points were fit by a logistic function (dashed lines). Note that the proportion of Δs differed in the two conditions to try to match task difficulty, with probabilities for Δs 10, 30, 50, and 70 Hz in the LRW condition 0.35, 0.28, 0.22, 0.15, and in the SRW condition 0.13, 0.21, 0.29, 0.37, respectively. **(B)** Rate of trial outcomes in the two conditions. **(C)** Proportion of high confidence reports on hit and miss trials as a function of change magnitude (Δ). **(D)** Proportion of high confidence reports on FA and CR trials. **(E)** Reaction times separated by Δ are plotted as the cumulative response probability as a function of the time from the change. Error bars indicate standard error of proportion.

The data points for the psychometric functions were generated from all change trials in which there wasn’t a FA; in other words, trials in which subjects were able to listen for a change. Proportion of ‘hits’ for a given Δ were plotted in Figure 2A, and data points were fit using a logistic function (Eq. 1).

(1) $$P\,\left(\Delta \right)=\frac{1}{1+10^{-\alpha \,\left(\Delta -\beta \right)}}$$

In Eq. 1, α corresponds to the slope and β corresponds to the 50% threshold of detection.

We computed the rates of the different trial types and expressed the proportion of FAs across all trials, the proportion of hits and misses across all change trials excluding trials in which there was a FA, and the proportion of CRs across all catch trials. The standard error for these proportions were determined using the *binofit* function in MATLAB (Figure 2B). Because confidence reports after each trial ended were binary (high confidence or low confidence), we similarly estimated the standard error for reporting high confidence in being successful for each trial outcome (i.e., hit, miss, FA, and CR) in Figures 2C,D.

##### Psychophysical reverse correlation detection report kernels

**FIGURE 3:**
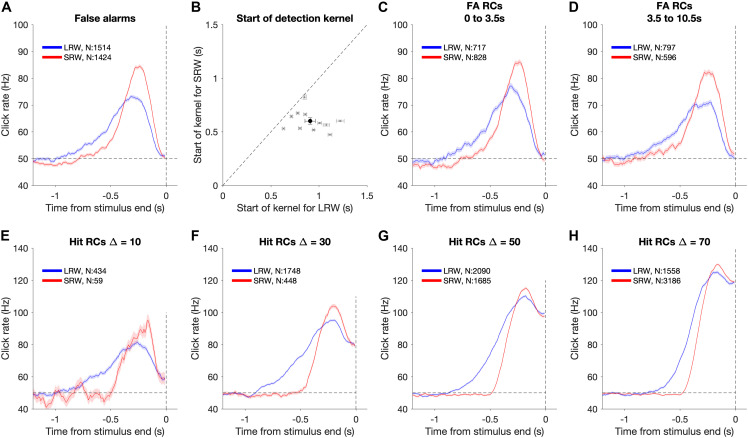
Using detection report kernels to compare the timescales of evidence evaluation between the task conditions. **(A)** Detection report kernel in the LRW (blue) and the SRW (red) conditions (data pooled across subjects, N shows number of trials). Shaded regions indicate the standard error of the mean. **(B)** Pairwise comparison of the start of detection report kernels between the task conditions. Gray points show individual subjects. Error bars for individual subjects indicate the standard error of the estimated start point. The black point shows the population average, and its error bar indicates the standard error of the mean across subjects. **(C)** Detection report kernels for false alarms occurring within the first 3.5 s of the trial. **(D)** Detection report kernels for false alarms occurring after 3.5 s from trial start. **(E–H)** Detection report kernels for hit trials with each panel for a different Δ. Same conventions as panel **(A)**.

We utilized psychophysical RC on FA trials to make model-free estimates of how subjects temporally weighted evidence to make their response decisions (Beard and Ahumada, 1998; Neri et al., 1999; Churchland and Kiani, 2016). RC “detection report kernels” were produced by aligning click trains from all FA trials of a given condition (i.e., LRW and SRW) to the times of the FAs (Figure 3A). The clicks were convolved using a causal half-gaussian filter (σ = 0.05 s) and averaged to produce detection report kernels, which estimate the average click rate that preceded a FA detection report. There was an attrition in the number of trials that contributed to each timepoint in the detection report kernel preceding the time of the FA due to the variability associated with the time of FA from the stimulus onset. Because FAs are defined as trials in which subjects responded during the baseline (pre-change) period and that stimulus is generated by a Poisson process, the actual rate of clicks calculated for any time interval during this period exhibits Poisson fluctuations around the mean generative rate. If subjects responded randomly, these fluctuations would average out to the mean generative rate. In contrast, if the RC kernel significantly differs from the generative baseline rate, it indicates that Poisson fluctuations in the actual click rate on individual trials influence the subjects’ detection responses. Furthermore, the Poisson property implies that there are no temporal correlations in the stimulus. Thus, deflections in the detection report kernel above baseline, if significant, reflect periods of time that influence the detection responses without being confounded by neighboring periods of time that may or may not have an influence.

To estimate the timescale of evidence evaluation for making detection responses, we estimated the start of the detection report kernel (Figure 3B). We fit the final 4 s of the detection report kernel until its maximum height using a two-piece linear function. This provided estimates and confidence intervals for three free parameters – baseline rate, slope, and the start point. To test whether the kernels were longer in the LRW compared to the SRW across subjects, we performed a pooled *t*-test on the mean difference in start points (H_0_: μ_LRW_ − μ_SRW_ ≤ 0).

To determine whether RCs depended on the time of the FA response, we also performed an identical analysis after first separating trials by the FA time (Figures 3C,D). We used two epochs, the first 3.5 s of the trial and greater than 3.5 s of the trial. To determine whether the RC differences between conditions were also present for hit trials, we performed the same analysis aligned to the time of the detection report on hit trials (Figures 3E–H). Hits necessarily involve the confounding influence of the increase in the generative stimulus rate, so an increase in the hit RC cannot be interpreted as readily as for FA trials. Nonetheless, we can still compare the hit RCs between conditions to find differences. These comparisons were based on estimates of the start of the detection report kernel in the same manner as described above for FA RCs.

##### Psychophysical reverse correlations confidence judgment kernels

**FIGURE 4:**
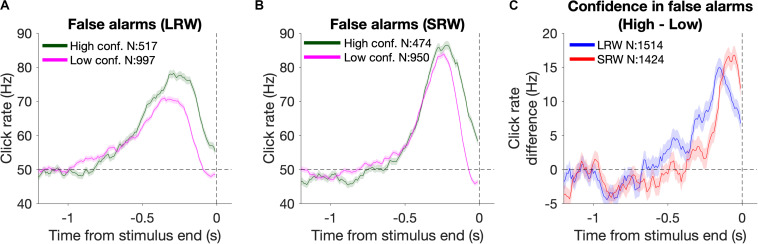
Changes in confidence evaluation timescales based on task condition. **(A,B)** Detection report kernels from the LRW and SRW conditions, respectively, sorted by the subsequent confidence judgment. **(C)** Confidence judgment kernels show the time periods when high confidence had a higher click rate than low confidence.

Reverse correlation analysis was also utilized to estimate the timescales of evidence evaluation for judging one’s confidence in successfully detecting a change (Figure 4). To do this, we generated detection report kernels as described above, but conditioned upon the subsequent confidence report. As high confidence was generally reported for FAs triggered by higher click rates, we estimated the time period over which the click rate was higher for high confidence FAs relative to low confidence FAs. This was done by taking the difference of the detection report kernels (high – low confidence) and approximating the start of this “confidence judgment kernel” using the same fitting method as described above for estimating the start of the detection report kernels.

#### Model-Based Analysis

We use the term “decision filter” to describe the internal temporal weighting function that subjects use for evidence evaluation. While RC-based detection report kernels provide information about how subjects temporally weight evidence, the precise decision filter can systematically differ from the detection report kernel (Okazawa et al., 2018). Thus, in addition to model-free methods to estimate timescales of evidence evaluation, we also utilized a model-based approach to estimate the decision filter. We used a six-parameter model that makes moment-by-moment probabilistic estimations of a decision variable (DV) that underlies decision commitment and the detection response. We then found the model parameters that maximized the likelihood of observing the experimental response times or lack of response (i.e., miss and correct reject trials involve no response) based on the stimuli subjects were presented on each trial. We used this to investigate how the decision filter changed when going from the LRW to the SRW condition.

**FIGURE 5:**
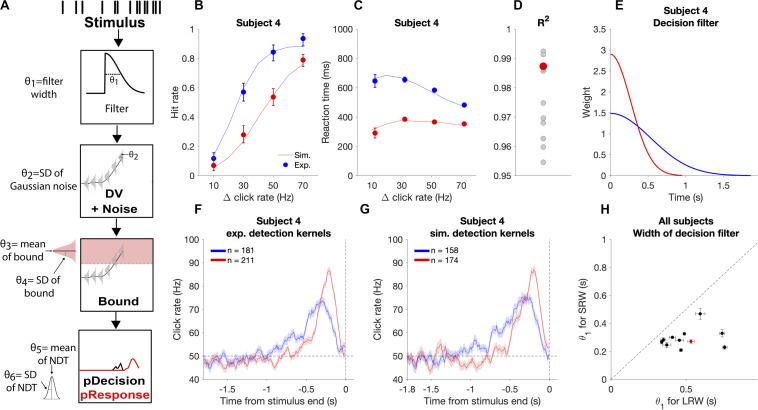
Modeling the decision process for the auditory change detection task. **(A)** Schematic of how the model estimates response time probabilities for single trials. Stimuli were convolved with a half-gaussian decision filter to generate the mean DV. Next, gaussian noise was added to the DV. The probability of decision commitment as a function of time was calculated based on the probability of crossing a bound with a variable height. Finally, a variable non-decision time is added to get the response time. **(B,C)** Experimental psychometric and chronometric data from subject four (data points) compared to model predictions using best fit parameters (lines). **(D)** Goodness of fit for each individual subject with subject four highlighted in red. **(E)** Changes in the best fit decision filter based on task condition for subject four, with a narrower filter in the SRW condition, **(F)** Experimental detection report kernels for subject four and **(G)** detection report kernels simulated from the best fit model for that subject with the same number of trials as the experimental data. **(H)** Pairwise comparison of the best fit decision filter width parameter (*θ*_1_) in the SRW vs LRW condition for each subject. Data from subject four is outlined by the red rectangle. Error bars indicate standard error of the estimated width parameter.

In the model, *θ* corresponds to a vector of model parameters (Figure 5A). *Step 1:* For each trial, the click times were convolved with a causal half-gaussian decision filter with width defined by the standard deviation of the gaussian (*θ*_1_). We utilized a causal filter to ensure that the current state of the DV was dependent only on past and not future clicks. This generated moment-by-moment estimates of the mean DV in the units of Hz. *Step 2:* Gaussian noise (*θ*_2_ = σ_DV noise_) was added to the DV to create a probabilistic distribution of the DV at each time step. *Step 3:* To determine the time of decision commitment, the model incorporated trial-to-trial decision bound variability, which was drawn from a gaussian distribution with *θ*_3_ = mean bound and *θ*_4_ = σ_bound_. *Step 4*: The weighted sum of the decision commitment times from step 3 was convolved with the gaussian NDT distribution with *θ*_5_ = mean NDT and *θ*_6_ = σ_NDT_. This yielded response time probability distributions for each trial.

We determined the likelihood of the parameters given the subjects responses in each trial. For hit and FA trials, the trial likelihood was calculated as the probability of the response time being within 10 ms of when the subjects actually responded. For miss and CR trials, the trial likelihood was the probability of not having responded for the full trial duration. The minimum trial likelihood value was set to 1/100,000 to provide robustness to any outlier response times (<1/260 trials). Using the Nelder-Mead simplex search algorithm, we determined the parameters that maximized the likelihood given the observed subjects’ responses by minimizing:

(2) $$l\,\left(\theta |X\right)=\sum_{i=1}^{N}-l\,o\,g\,\left(L\,\left(\theta |x_{i}\right)\right),$$

where *θ* is the set of parameters, *X* are the observed responses across all trials within a condition, *i* is the trial number, *N* is the number of trials in the condition, and *L*(*θ*| *x*_i_) is the parameters’ likelihood given the response in trial *i*.

The standard error of the parameter estimates were obtained by calculating the hessian matrix (H) for the point *l*(*θ*| *X*) and calculating $\sqrt{d\,i\,a\,g\,\left(H^{-1}\right)}$. The data did not provide well-constrained estimates with DV noise as a free parameter, so we fixed this parameter to the median value found across all subjects and task conditions. Differences in decision filters between conditions were robust to different values of fixed DV noise. Goodness of fits were calculated using *R*^2^ to quantify the fraction of the variance of the reaction time (chronometric) and hit rate (psychometric) functions explained by the model.

## Results

### Task Performance

A total of 11 subjects were trained on the auditory change detection task (see section “Materials and Methods” for details). In brief, each trial began with a series of clicks generated by a 50 Hz Poisson process. Subjects were trained to respond when they perceived an increase in the generative click rate. The change magnitude (Δ) was randomly chosen for each change trial, which served as a way to vary trial difficulty. A total of 30% of trials were catch trials (randomly interleaved), meaning they contained no change in the generative click rate and the appropriate action was to withhold a response until the stimulus terminated.

All subjects quickly learned the detection task design and performance plateaued within two training sessions. Subjects performed 3–5 testing sessions of the LRW version followed by 3–5 testing sessions of the SRW versions of the task, which corresponded to a median of 1,347 trials per condition per subject (range: 774 to 2,209 trials). To match overall task difficulty between conditions, the low magnitude Δs (10 and 30 Hz) were more prevalent in the LRW, while the high magnitude Δs (50 and 70 Hz) were more prevalent in the SRW.

Similar to previous results (Johnson et al., 2017; Ganupuru et al., 2019), hit rates were low for the most difficult trials (Δ = 10 Hz) and progressively increased for larger Δs (Figure 2A). Subjects’ performance depended upon the task condition (*F*(3, 80) = 25.91, *p* = 8.24e-12 for the Δ-condition interaction, ANOVA), such that subjects had lower hit rates in the SRW condition for each Δ (*p* < 0.001 for all Δs). Intuitively, subjects had lower hit rates in the SRW condition because they had a shorter time to respond to changes. As subjects went from the LRW to the SRW condition, there was a mean decrease in the slope (0.051 ± 0.001 vs 0.032 ± 0.001; *p* < 0.001) and an increase in detection threshold (26.5 ± 1.2 vs 49.6 ± 2.2; *p* < 0.001) as calculated from psychometric functions of individual subjects. There was a 37.8% ± 10.1% decrease in the slope and 86.0 ± 6.1% increase in the threshold in the combined subject data (dashed lines in Figure 2A).

False alarm rates were under 10% and similar in both conditions, and miss rates were 43 and 51% in the LRW and SRW conditions, respectively (Figure 2B). The similar error rates across conditions was not surprising because the task was designed to control for difficulty across conditions by including a greater proportion of low magnitude Δs in the LRW condition and a greater proportion of high magnitude Δs in the SRW condition. While subjects clearly had higher hit rates for each Δ in the LRW condition, the number of trials that contributed to each data point in Figure 2A were uneven across Δs and conditions. CR rates were similar for both conditions, around 90%.

At the end of each trial, subjects reported either high or low confidence in successfully completing the trial by poking either the right or left port, respectively. In hit trials, subjects’ confidence progressively increased for increasing Δs (Figure 2C). In miss trials, subjects’ confidence progressively decreased for increasing Δs in the SRW condition. The trend was not as clear in the LRW condition, but miss trials were rare for the higher Δs in this condition. In both conditions, the proportion of high confidence reports in FA trials was lower than in hit trials, and the proportion of high confidence reports in CR trials was higher than in miss trials (Figure 2D).

### Subjects’ Reaction Times Were Faster in the SRW Condition

In addition to examining changes in psychometric functions, we also examined changes in reaction times. To examine reaction times across conditions with different response windows, we plotted the cumulative response probability as a function of the time from the change for each Δ in the two conditions (Figure 2E). Subjects responded faster to changes in the SRW condition (steeper slope of the traces). By 500 ms, the cumulative response probability was different across task conditions (*F*(3, 80) = 3.61, *p* < 0.05), such that the cumulative response probability at 500 ms was higher for all Δs in the SRW condition (*p* = 0.022, <0.01, <0.005, <0.001 for Δ = 10, 30, 50, and 70, respectively). While the faster responses in the SRW could be due to subjects utilizing shorter timescales of evidence evaluation in this condition, other mechanisms like shortened sensorimotor delays (i.e., NDTs) could also explain the faster reaction times in the SRW. We note that if there was purely a decrease in the decision bound when switching to the SRW condition, we would expect to see faster reaction times but also higher FA rates. However, as previously mentioned, FA rates were similar across conditions.

### Psychophysical Reverse Correlation Demonstrates That Subjects Adaptively Adjusted Their Timescale of Evidence Evaluation for Detection Reports

We next investigated if subjects were indeed utilizing shorter timescales of evidence evaluation in the SRW compared to the LRW condition to make their detection reports. To do this, we estimated the timescales of evidence that had leverage on subjects’ detection reports using psychophysical RC similar to previous studies to estimate detection report kernels (Johnson et al., 2017; Ganupuru et al., 2019). Detection report kernels can be used to infer how subjects temporally weight evidence for a detection report. The RC analysis was specifically focused on FAs, which are defined as responses that occurred during the noise-only period of trials when the generative click rate was 50 Hz. Even though the generative click rate is 50 Hz, there are random fluctuations of the actual click rate in small windows of time due to the Poisson nature of the stimulus. If subjects were randomly responding, these fluctuations would even out on average and detection report kernels would approximate a horizontal line at 50 Hz, the baseline generative click rate. In contrast, a significant upward deflection above baseline in the detection report kernel indicates that subjects were on average responding to local increases in the fluctuations of the click rate over that period of time.

Figure 3A shows the shape of the detection report kernels in LRW and SRW conditions (data pooled across subjects). Detection report kernels in both conditions exhibited a clear increase prior to the response, indicating that subjects’ FAs were on average preceded by a period of increased click rates. Detection report kernels in both conditions returned to the 50 Hz baseline shortly before the choice, consistent with clicks during this period being too late to have leverage on the response, matching previous observations (van den Berg et al., 2016; Johnson et al., 2017; Ganupuru et al., 2019). The amplitude of the detection report kernel in the SRW condition was significantly higher than in the LRW condition, indicating that subjects were responding to relatively higher instantaneous click rates in the SRW condition. However, the width of the detection report kernel was narrower in the SRW condition, indicating that a relatively shorter period of evidence had leverage on subjects’ FA choices in the SRW condition. We fit the detection report kernel of each subject in the two conditions with a piecewise linear function to estimate its start time. The pairwise comparison of detection report kernel start times is summarized in Figure 3B. Points to the right of the diagonal line are indicative of longer detection report kernels in the LRW condition, an effect found to be significant in our population (*p* < 0.001). On average, the detection report kernel started 308 ± 21 ms later in the SRW condition, suggesting that subjects adaptively adjusted their timescale of evidence evaluation for their detection reports depending on task demands.

The difference in detection report kernels between conditions was not due to a confound of time of the decision response. While the distribution of change times was identical between conditions, the times of FA responses that are used for the detection report kernel analysis depends on the subject’s behavior. Thus, it is possible that differences in detection report kernels between conditions may reflect differences in the timing of the FA response. To address this, we separated FA trials based on whether the FA occurred within the first 3.5 s of the trial or greater than 3.5 s from trial start and compared detection report kernels between the two conditions separately for each epoch (Figures 3C,D). In both epochs, the main result held: detection report kernels were significantly longer in the LRW condition (*p* < 0.001), with the kernels starting on average 298 ± 24 ms later in the SRW condition for the earlier epoch and 340 ± 19 ms later in the SRW condition for the later epoch.

The results also extended to analyses that considered other trial types in addition to FAs. In particular, hit trials also revealed similar differences in timescales of evidence evaluation between the LRW and SRW conditions as FA trials. Because hit trials necessarily involve an increased click rate that depends on the Δ, we performed the psychophysical RC analysis separately for each (Figures 3E–H). For all Δs, we found that the detection report kernel on hits was significantly longer in the LRW condition (*p* < 0.01 in all cases), with the kernels starting on average 355.2 ± 22.1, 345.5 ± 8.3, 248.8 ± 5.6, and 174.8 ± 5.5 ms later in the SRW condition for Δs of 10, 30, 50, and 70, respectively.

### Psychophysical Reverse Correlations Demonstrate That Subjects Adaptively Adjusted Their Evaluation Timescale for Confidence Reports

In addition to reporting change detections, subjects also reported their confidence in successfully completing each trial. Confidence reports thus present another opportunity to investigate how evidence evaluation timescales change based on task demands, and whether those changes are consistent between different aspects of decision reports. To examine this, we again used detection report kernels, but this time they were generated based on whether subjects subsequently reported high or low confidence. The detection report kernels from the combined subject data sorted by confidence from the LRW and SRW conditions are presented in Figures 4A and B, respectively. Subjects generally reported high confidence when there was more evidence of a change (area under the detection report kernels). Unlike for FA reports (Figure 3A), confidence judgments on FA trials were informed by evidence until the end of the stimulus, as shown by the separation in the high and low confidence detection report kernels at the end of the stimulus. This occurred because confidence judgments were reported after the time of the detection response, and therefore, subjects had additional time to incorporate evidence immediately preceding their detection responses, similar to previous studies (van den Berg et al., 2016; Ganupuru et al., 2019).

The time period over which detection report kernels were higher in the high confidence trials compared to the low confidence trials provides information about the time period that had positive leverage for judging confidence as high versus low. We determined the start of this period by fitting the confidence judgment kernel (high – low confidence detection report kernels) with a two-piece linear function to estimate when it inflected upward starting from a 0 Hz difference. We found that this time period was narrower in the SRW condition compared to the LRW condition (272 ± 16 ms vs 572 ± 35 ms, respectively) (Figure 4C), suggesting that the timescales for evaluating confidence in detection trials adapted to task demands as well.

### Model-Based Analysis Reveals Subjects Contextually Adapted Their Timescales of Evaluation

While RC analyses are a powerful model-free approach to examine the timescale of evidence evaluation, they do not provide a perfect reflection of the decision filter through which evidence is temporally processed by the brain for a decision (Okazawa et al., 2018). Therefore, we also estimated how the decision filter changed based on the task condition using a model-based approach.

We applied a behavioral model that makes moment-by-moment probabilistic estimates of a DV for a response based on the evaluation of sensory evidence. This model estimates the distribution of reaction times given a particular stimulus and set of model parameters. We fit this model to the data separately for the SRW and LRW conditions to compare best fit parameters in each condition for each subject.

We validated that the estimated parameters provide reasonable accounts of individual subjects’ behavioral performance by simulating task responses using the subject-condition specific parameter estimates and new sets of stimuli. We found that simulations closely approximated subjects’ experimental psychometric functions, chronometric functions, and detection report kernels (Figure 5), thus offering face validity of the behavioral model. Fits were performed on data from individual subjects, as illustrated in Figure 5, and had similar goodness of fit across all subjects with *R*^2^ above 0.95 for all subjects (Figure 5D).

We used this approach to examine if the decision filter adapted to the task condition by becoming narrower when subjects went from the LRW to the SRW condition. We found a mean decrease in the decision filter width in the SRW condition (σ = 0.50 ± 0.07 s vs 0.29 ± 0.06 s in the LRW vs. SRW, respectively, *p* < 0.005). Moreover, the estimated decision filter was narrower in the SRW condition for all subjects (Figure 5H; *p* < 0.005 for all comparisons). Thus, the model-based approach corroborates our model-free findings showing that subjects indeed adapted their evaluation timescales for detecting changes in order to match task demands.

Table 1 summarizes the estimated model parameters for each subject. We note that the estimates of the bound also increased for all subjects in the SRW condition (*p* < 0.005), which we discuss below. We did not find any significant changes in the mean NDT parameters nor in the trial-to-trial bound variability across conditions.

**TABLE 1:** Subject-specific model parameter estimates ± SE for each condition (LRW, long response window; SRW, short response window). Subjects identified by number.

	**Filter width**	**Mean NDT**	**σ NDT**	**Mean bound**	**σ Bound**
LRW 1	0.37 ± 0.028	0.21 ± 0.013	0.07 ± 0.010	94.79 ± 1.454	8.560.555
LRW 2	0.74 ± 0.017	0.16 ± 0.011	0.08 ± 0.013	77.39 ± 0.468	5.830.407
LRW 3	0.49 ± 0.008	0.17 ± 0.005	0.03 ± 0.005	84.31 ± 0.413	4.680.336
LRW 4	0.53 ± 0.025	0.20 ± 0.009	0.06 ± 0.011	81.87 ± 0.874	6.180.430
LRW 5	0.34 ± 0.007	0.18 ± 0.005	0.05 ± 0.005	87.36 ± 0.475	5.710.377
LRW 6	0.76 ± 0.016	0.14 ± 0.009	0.06 ± 0.012	78.32 ± 0.448	5.920.357
LRW 7	0.35 ± 0.017	0.19 ± 0.006	0.05 ± 0.004	89.40 ± 0.925	5.810.402
LRW 8	0.59 ± 0.032	0.16 ± 0.008	0.05 ± 0.008	78.98 ± 0.807	5.140.347
LRW 9	0.46 ± 0.028	0.28 ± 0.012	0.07 ± 0.007	90.57 ± 1.150	7.450.432
LRW 10	0.47 ± 0.009	0.22 ± 0.007	0.06 ± 0.005	86.47 ± 0.505	7.350.367
LRW 11	0.41 ± 0.020	0.23 ± 0.008	0.07 ± 0.007	90.26 ± 1.022	9.640.460
Mean LRW:	0.50 ± 0.069	0.19 ± 0.029	0.06 ± 0.028	85.43 ± 2.797	6.571.363
SRW 1	0.25 ± 0.019	0.21 ± 0.009	0.07 ± 0.005	98.16 ± 1.859	7.640.775
SRW 2	0.33 ± 0.026	0.17 ± 0.009	0.04 ± 0.007	96.90 ± 2.156	10.130.887
SRW 3	0.33 ± 0.005	0.17 ± 0.003	0.03 ± 0.003	86.35 ± 0.408	4.700.351
SRW 4	0.27 ± 0.014	0.17 ± 0.005	0.05 ± 0.003	89.85 ± 1.096	6.560.472
SRW 5	0.27 ± 0.013	0.18 ± 0.005	0.04 ± 0.003	91.91 ± 1.101	6.150.450
SRW 6	0.23 ± 0.015	0.16 ± 0.006	0.05 ± 0.004	96.22 ± 1.493	7.590.565
SRW 7	0.28 ± 0.015	0.19 ± 0.005	0.04 ± 0.003	91.51 ± 1.196	5.830.468
SRW 8	0.47 ± 0.040	0.14 ± 0.009	0.04 ± 0.007	81.79 ± 1.351	4.540.532
SRW 9	0.28 ± 0.005	0.21 ± 0.004	0.04 ± 0.003	95.77 ± 0.666	7.760.464
SRW 10	0.21 ± 0.009	0.19 ± 0.005	0.04 ± 0.003	100.65 ± 1.18	9.370.501
SRW 11	0.30 ± 0.007	0.24 ± 0.007	0.07 ± 0.006	95.13 ± 0.947	8.450.656
Mean SRW:	0.29 ± 0.060	0.19 ± 0.021	0.05 ± 0.015	93.11 ± 4.349	7.161.912

## Discussion

To investigate the flexibility of evidence evaluation timescales, we utilized an auditory change detection task and varied the allotted window to respond to a change. Because the baseline click rate was fixed at 50 Hz, the normative strategy for an ideal observer in this task involves integrating evidence over a timescale commensurate with the response window and setting a decision bound on the accumulated evidence that would minimize the total error rate (i.e., misses and FAs). In this way, the ideal observer would capture all the relevant evidence of a real change in order to maximize the discriminability between signal and noise. Owing to inherent sensorimotor delays, the normative strategy for a real observer involves accumulating evidence over a timescale slightly shorter than but proportional to the response window and similarly setting the decision threshold to minimize total error rates. In this report, we demonstrated that subjects utilized narrower evaluation timescales for SRW compared to LRW conditions.

Our results provided additional support for the idea that temporal weighting of evidence is flexible and adaptive to task demands. Previous studies have shown that a variety of species adapt their temporal weighting of evidence based on task demands. Humans and monkeys adopt temporal weighting of evidence to match learned temporal regularities of stimuli that are repeatedly presented, giving more weight to time periods with higher fidelity information (Levi et al., 2018). In that study, visual stimuli were presented for a fixed amount of time with no underlying variability in the time at which the information fidelity changed. In that situation, a temporal weighting function can potentially be anchored to an external cue, such as stimulus onset. In environments that involve change points that vary in timing, the temporal weighting function should instead shift as time elapses so that the most weight is given to the most recent times. This describes what we call a “decision filter” that sets the timescale of evidence evaluation, with an optimal timescale that depends on the statistics of the environmental changes (Glaze et al., 2015; Radillo et al., 2017; Piet et al., 2018). In particular, more volatile environments demand shorter timescales. Both humans and rats have been shown to adapt their timescales of evidence evaluation in the optimal direction as a function of volatility when discriminating the current state of a sensory stimulus in a changing environment (Glaze et al., 2015; Piet et al., 2018). In those studies, the tasks did not require explicit detection of the change point. Instead, the decision filter applies equally regardless of whether a change occurred or not. While similar considerations of optimal timescales of evidence evaluation extend to our task, it differs in requiring the detection of the change point itself.

Our primary manipulation involved the signal duration after a change, with a limited window of time that differs between conditions to respond to changes. Failure to respond within that time resulted in trial termination. This is similar to many natural situations where failure to respond quickly to an environmental change can have immediate negative consequences, such as predator avoidance, optimal foraging behavior (Kilpatrick et al., 2020), or specifically for humans, driving a vehicle. Previous work has shown the timescales of evidence evaluation can be adapted to expected signal durations when humans perform a visual change detection task (Ossmy et al., 2013). Our work extends these results in three primary ways. First, in addition to using model-based analyses similar to the previous study, we corroborate the main finding using model-free psychophysical RC methods not used in the previous study. Second, while the previous work involved visual detection, our work involves detecting an auditory signal in noise, one of the fundamental functions of the auditory system (Bronkhorst and Plomp, 1992; Narayan et al., 2007; Shetake et al., 2011; Moore et al., 2014; Christison-Lagay et al., 2017) and suggests another important role for contextual modulation of sound processing (Angeloni and Geffen, 2018). Third, our task also involves a confidence judgment component (Ganupuru et al., 2019), allowing us to show that the timescale of evidence evaluation for confidence in the detection responses also adapts to expected signal durations (as discussed further below).

In addition to the changes in the timescale of evidence evaluation between task conditions, there were also changes in the model-based estimates of the bound between conditions, with a higher bound for a short response window (Table 1). We suspect this occurred for two reasons. First, subjects may have learned to expect generally higher Δs in the SRW condition, as this was a feature of the task that we intentionally designed to yield similar error rates across conditions (see section “Materials and Methods” for details). Second, subjects evaluated evidence over shorter timescales in the SRW condition and this introduces more variance in the DV that is based on the evaluated evidence. An increase in the bound in the SRW condition as we observed would help to curtail an increase in the FA rate that would result from maintaining the same bound when evaluating evidence over shorter timescales. We note that we made the design choice to try to match overall task difficulty between conditions, which required different proportions of change magnitudes. Importantly, the optimal width of the decision filter is not affected by the proportion of change magnitudes. In our task design, a missed change results in immediate ending of the trial after the response window duration, so the ideal observer should not evaluate evidence further into the past. Any evidence from the more distant past than the duration of the response window necessarily precedes a change in this task. In addition, faster decision responses result in no speeding of the trial durations, so there is no advantage conferred for evaluating evidence in a briefer period than what is allowed given the response window and considering the NDT. Evaluating over the longest duration given those constraints maximizes performance regardless of the stimulus strengths presented.

By incorporating confidence reports into the task design, we were able to obtain an independent measure of how evidence evaluation timescales varied based on task demands. Similar to the detection reports, confidence in the detection report should also be proportional to the response window in order to maximize performance. On detection trials, a feature of the evidence that should yield high confidence is a relatively high click rate over a period of time preceding the response. We found that this time period was shorter in the SRW compared to the LRW condition (Figure 4), suggesting that subjects contextually adapted their confidence evaluation timescales as well. While the nature of the effect was similar for the confidence judgment as the detection report, the confidence judgment was reported after detection. Consistent with previous results using a similar design, the period of time influencing the latter is offset from that influencing the former. Therefore, we did not incorporate the confidence judgment into our model for this task. We also note that our results fully replicated previous work from our lab showing flexibility in the timescales of evidence evaluation when comparing detection decisions and associated confidence judgments (Ganupuru et al., 2019).

While any timepoint shown to have a RC kernel significantly greater than baseline necessarily has positive leverage on the detection report on average, it is also possible that timepoints without a significant effect also have positive leverage. The RC kernels reflect a complex interplay between how evidence is temporally weighted through the decision filter, bound, and NDT. Moreover, RC analyses introduce sampling bias related to evaluating only epochs that preceded a response during pre-signal periods of stimuli (Okazawa et al., 2018). Model-based analyses provide a complementary approach to estimate the decision filter. This approach explicitly estimates the effects of other factors such as decision bound and NDT, so it potentially provides a way to overcome the challenges those factors pose for interpreting RC kernels. For this reason, we also utilized the model-based approach to estimate how the decision filter changed based upon task condition, with results that confirmed our RC kernel findings. These are complementary approaches, with the modeling potentially able to distill separate components of the decision process, but in a way that depends on the quality of the model. Even though the goodness of the model fits were consistently high across subjects, it is reassuring to have similar results about the decision filter from the model-free RC kernel analysis that does not depend on the quality of the model.

For the model, all trial types were utilized to estimate six model parameters; however, well-constrained estimates were not achievable when DV noise was treated as a free parameter, and thus we constrained DV noise to 6 Hz (the median DV noise value across all subject using the unconstrained approach). We additionally estimated parameters by constraining DV noise to values ranging from 3 to 9 Hz and found that while the nature of difference in decision filter width between conditions was preserved, it systematically increased or decreased the width, respectively, across conditions. Thus, while we cannot precisely estimate the decision filter for these data, we can conclude that the decision filter was relatively narrower in the SRW condition, and thus adapted to the context.

The neural mechanisms of evidence evaluation for decision making have been studied in large part using paradigms where the optimal strategy involves perfect integration of evidence (Brody and Hanks, 2016). These studies have revealed many brain regions with signals related to evidence evaluation (Gold and Shadlen, 2007; Brody and Hanks, 2016; Hebart et al., 2016). However, it is not yet known how these regions are involved in evidence evaluation across different timescales nor what neural mechanisms control those timescales. Answering these questions is an important goal for future research.

## Data Availability Statement

The datasets generated for this study are available on request to the corresponding author.

## Ethics Statement

The studies involving human participants were reviewed and approved by UC Davis Institutional Review Board. The patients/participants provided their written informed consent to participate in this study.

## Author Contributions

RH, EJ, HP, and TH conceived and designed the experiment. RH, EJ, and HP collected the data. RH analyzed the data with help from PG. RH, PG, AG, and TH interpreted the results and edited the manuscript. RH and TH drafted the manuscript with input from PG and AG. All authors contributed to the article and approved the submitted version.

## Conflict of Interest

The authors declare that the research was conducted in the absence of any commercial or financial relationships that could be construed as a potential conflict of interest.
